# Estimating the cost due to resistance against antiretroviral therapies in individuals with HIV: Perspective of the Kingdom of Saudi Arabia

**DOI:** 10.1016/j.ijregi.2024.100371

**Published:** 2024-04-25

**Authors:** Wali Ghassan, Alraddadi Basem, Albayat Hawra, Alharbi Ahmad, Abdulrahman Ahmed Hasan Muaddi, Asma Mestouri, Rezk Elaraby, James Mahon

**Affiliations:** 1 King Faisal Specialist Hospital & Research Centre, Jeddah, Saudi Arabia; 2 King Saud Medical City, Riyadh, Saudi Arabia; 3 National Guard Health Affairs, Riyadh, Saudi Arabia; 4 Prince Sultan Military Medical City, Riyadh, Saudi Arabia; 5 Gilead, Dubai, United Arab Emirates; 6 Coldingham Economics, United Kingdom

**Keywords:** Antiretroviral therapy, Drug resistance, Cost burden, Kingdom of Saudi Arabia

## Abstract

**Objectives:**

The emergence of resistance to antiretroviral therapy (ART) has an impact on the cost of HIV care. This study aimed to estimate the direct and indirect costs associated with the first episode of drug resistance in individuals with HIV receiving first-line ART.

**Methods:**

We developed a cost calculator to estimate the cost of drug resistance over a period of 12 months in the Kingdom of Saudi Arabia. The model inputs (estimated using expert opinion and publicly available sources) included costs associated with testing for resistance, adverse events of a new regimen, and indirect costs.

**Results:**

The direct and indirect medical expenses for the year resistance developed were 6980 Saudi Arabian riyal (SAR) and SAR 2862, respectively. The addition of the cost of new ARTs would increase the total annual costs (between SAR 5174 and SAR 34,265 per patient). One-way sensitivity analysis also reported significant impact of initial and switching therapies used after resistance develops on the total costs of resistance per year.

**Conclusions:**

There is a significant cost burden associated with drug resistance, which emphasizes the need to select an appropriate initial ART regimen that has a strong genetic barrier and conduct pre-treatment resistance tests (if possible).

## Highlights

• Drug resistance to antiretroviral therapy has a significant cost burden.
• A cost calculator was designed to estimate the cost per episode in the Kingdom of Saudi Arabia.
• The annual costs varied for patients due to variable antiretroviral therapy switch cost.
• Initiation with antiretroviral therapy having a high genetic barrier reduces resistance and cost burden.

## Introduction

HIV infection remains a challenge worldwide despite the medical and technological advancements in the last 2 decades [1]. About 37.5 million adults and 1.2 million children (<15 years old) were had HIV infection worldwide in 2022, resulting in a global prevalence of 39.0 million, with a higher prevalence in women [2]. An increasing number of HIV cases have been observed in the Middle East and North Africa, with 180,000 people living with HIV infection as per 2021 data [3]. In the Kingdom of Saudi Arabia (KSA), the Ministry of Health has implemented surveillance for HIV infections since the first case of HIV was registered in 1984 [4].

Antiretroviral therapy (ART) is recommended as the preferred choice of therapy by different international bodies for the treatment of patients with HIV [[5], [6], [7]]. According to the World Health Organization (WHO) report 2022, approximately 29.8 million patients with HIV worldwide were under ART treatment [5]. However, in recent years, the widespread use of ART has resulted in an increased prevalence of HIV drug resistance [8], further escalating the cost of HIV management [9,10].

The resistance to HIV drugs often limits the therapeutic options for HIV management, compelling physicians to shift to advanced treatment regimens, which may have unwanted side effects, higher pill burden, increased chances of drug interactions, and increased medical cost [9]. Furthermore, HIV drug resistance may worsen long-term clinical outcomes [9,11]. Therefore, routine use of drug resistance testing can improve survival outcomes, reducing the long-term cost of HIV management [9,10,12].

Health-economic studies evaluating the impact of HIV drug resistance on HIV management and the overall health care budget are limited, and no such studies have been conducted in KSA to the best of our knowledge. Hence, the current study was conducted to calculate the cost associated with an initial episode of drug resistance in patients with HIV who were on first-line ART from a national perspective in KSA.

## Methods

### Model structure and methodology

This Microsoft Excel–based cost calculator was designed to estimate the cost of an episode of drug resistance in an individual infected with HIV who exhibits virological failure on first-line ART in KSA. The regimens recommended as first-line ARTs by WHO and European and US guidelines were studied and validated by experts for model inputs in this study [[5], [6], [7]]. First-line ARTs considered in the model are lamivudine/dolutegravir, dolutegravir/lamivudine/abacavir, dolutegravir + tenofovir alafenamide/emtricitabine, and elvitegravir/cobicistat/emtricitabine/tenofovir alafenamide, which were based on available guidelines and validated by experts [[5], [6], [7]]. Patient were switched to new ARTs when they developed resistance, which included bictegravir/emtricitabine/tenofovir alafenamide, tenofovir alafenamide + ritonavir + darunavir + dolutegravir, and darunavir + tenofovir alafenamide/ emtricitabine +ritonavir, as suggested by the guidelines and validated by experts [[5], [6], [7]].

### Model settings

The model considered a societal perspective and a time horizon of 1 year for costs associated with direct and indirect expenses. The key features of the model are presented in Supplementary Table S1.

### Study population

Adult population infected with HIV and experiencing the first episode of drug resistance while on initial ART were considered for this model. The model estimated the annual cost of treatment per patient due to an episode of drug resistance. The proportion of patients switching to these therapies are shown in Supplementary Table S2. Patients with secondary or acquired resistance were not included in this analysis.

### Model inputs

As per expert opinion, the patients were assumed to require two additional clinic visits for resistance tests. The costs of these visits (Table 1) and resistance tests (Table 2) were obtained from five experts, which included four clinicians and one payer.

**Table 1:** Costs per person due to additional clinic visits to manage AEs.

AEs	Type of clinician treating AE	Number of contacts until resolved	Cost per visit (Saudi riyals)	Source
Headache	HIV physician	3	150	KOL
Dizziness	HIV physician	2	150	KOL (assumption on the number of visits)
Peripheral neuropathy	Neurologist	10	150	KOL (assumption on the number of visits)
Nausea	HIV physician	3	150	KOL (assumption on the number of visits)
Diarrhea	HIV physician	3	150	KOL (assumption on the number of visits)
Back pain	HIV physician	3	150	KOL (assumption on the number of visits)
ALT elevation	Hepatologist	5	150	KOL (assumption on the number of visits)
AST elevation	Hepatologist	5	150	KOL (assumption on the number of visits)
Fatigue	General practitioner	5	75	KOL (assumption on the number of visits)
Amylase elevations	Gastroenterologist	5	150	KOL (assumption on the number of visits)
Gastrointestinal	Gastroenterologist	5	150	KOL (assumption on the number of visits)
Alanine aminotransferase	Hepatologist	5	150	IQVIA Insight
Aspartate aminotransferase	Hepatologist	5	150	IQVIA Insight
Total cholesterol	Endocrinologist	6	150	IQVIA Insight
LDL elevation	Endocrinologist	6	150	IQVIA Insight
Hyperglycemia	Endocrinologist	5	150	IQVIA Insight
Pancreatic amylase	Hepatologist	6	150	IQVIA Insight
Triglycerides	Endocrinologist	6	150	IQVIA Insight
Arthralgia	General practitioner	3	75	IQVIA Insight
Chlamydial infection	General practitioner	2	75	IQVIA Insight
Upper respiratory tract infection	General practitioner	2	75	IQVIA Insight
Creatine kinase concentration elevation	HIV physician	5	150	IQVIA Insight
Serum glucose concentration elevation (fasting hyperglycemia)	Endocrinologist	4	150	IQVIA Insight

AEs, adverse events; KOL, key opinion leader; LDL, low-density lipoprotein; SAR, Saudi riyal.

**Table 2:** Costs per person for resistance tests.

Type of tests	Proportion of patient receiving tests^a^	Cost per test (Saudi riyals)^b^
**Genotype/phenotype tests performed when resistance suspected**		
Protease inhibitor/NRTI/NNRTI genotype resistance test	100%	1500
INSTI genotype resistance test	100%	3000
INSTI phenotype resistance test	0%	2000
EI phenotype resistance test	0%	2000^c^
Co-receptor tropism test for C-C chemokine receptor type 5 screening	0%	7330^d^
**Tests performed when new ART started, number of tests required**		
Metabolic panel test, 4	100%	120
Cell blood count, 4	100%	80
White blood cell count, 4	100%	80
Electrocardiography, 1	5%	100
Electroencephalography, 1	1%	400
Cardiac stress test (treadmill or dobutamine), 1	2%	1,500
Fasting Blood Glucose, 3	70%	120
Glycated hemoglobin, 2	70%	80
Renal function test (blood urea, serum creatinine), 4	100%	120
Phycologist/psychiatrist, 1	10%	150

INSTI, integrase strand transfer inhibitor; NA, not applicable; NRTI, nucleoside reverse transcriptase inhibitor; NNRTI, non-NRTI.

a Based on expert opinions;

b all costs except the cost of Co-receptor tropism test for CCR5 screening of were obtained from expert opinions;

c assumed to be same as INSTI phenotype test;

d McCarthy et al. Use of a Commercial HIV Co-Receptor Tropism Assay in Clinical Practice, 2015.

The total annual costs of all initial therapies and switched therapies were calculated based on the prices obtained from the Saudi Food and Drug Authority (Supplementary Table S3).

The rates for adverse events (AEs) were obtained from the literature. The dosage and duration of drugs used to treat AEs were obtained from expert interviews and the costs for drugs were retrieved from key opinion leader inputs and the Saudi Food and Drug Administration (Supplementary Tables S4 and S5).

For indirect costs, the societal value of quality-adjusted life years (QALYs) was calculated based on the cost-effectiveness threshold values per QALY suggested by Al-jedai et al. [13]. The anxiety utility decrement was obtained from Kind et al. [14] and the duration of anxiety was assumed to be 8 weeks, which is the time it takes for the viral load to fall back to target. The productivity losses were calculated based on expert opinion (Table 3).

**Table 3:** Breakdown of indirect costs and their sources.

Parameter	Value	Source
**Societal value of QALY**		
Value per QALY	SAR 62,500	Al-jedai et al. [13]
**QALY loss due to resistance**		
Anxiety utility decrement per person	0.08	Kind et al. [14]
Duration of anxiety (weeks)	8	Assumed to be the 8 weeks until viral load falls back to target
Total QALY loss due to resistance	0.01	Calculation
**Productivity loss**		
Daily wage (SAR)	422	World Bank (Assumed 42 working weeks per year with 5 days per week)^a^
Days lost from resistance testing	2	Key opinion leader
Days lost from adverse events	3	Assumption

QALY, quality-adjusted life-year; SAR, Saudi riyal.

a World Bank (available: https://data.worldbank.org/country/saudi-arabia. accessed on Dec 29, 2022).

### Analytical approach

The model calculated total costs per patient due to resistance, which included direct and indirect costs. The direct health care costs included additional ART, additional clinic visits, drug resistance tests, additional clinical tests, and AEs from switching to new ART. The indirect societal costs included QALY loss from resistance-based anxiety and loss in productivity due to loss of daily wage and additional days lost for additional clinical visits and due to AEs.

**Figure 1:**
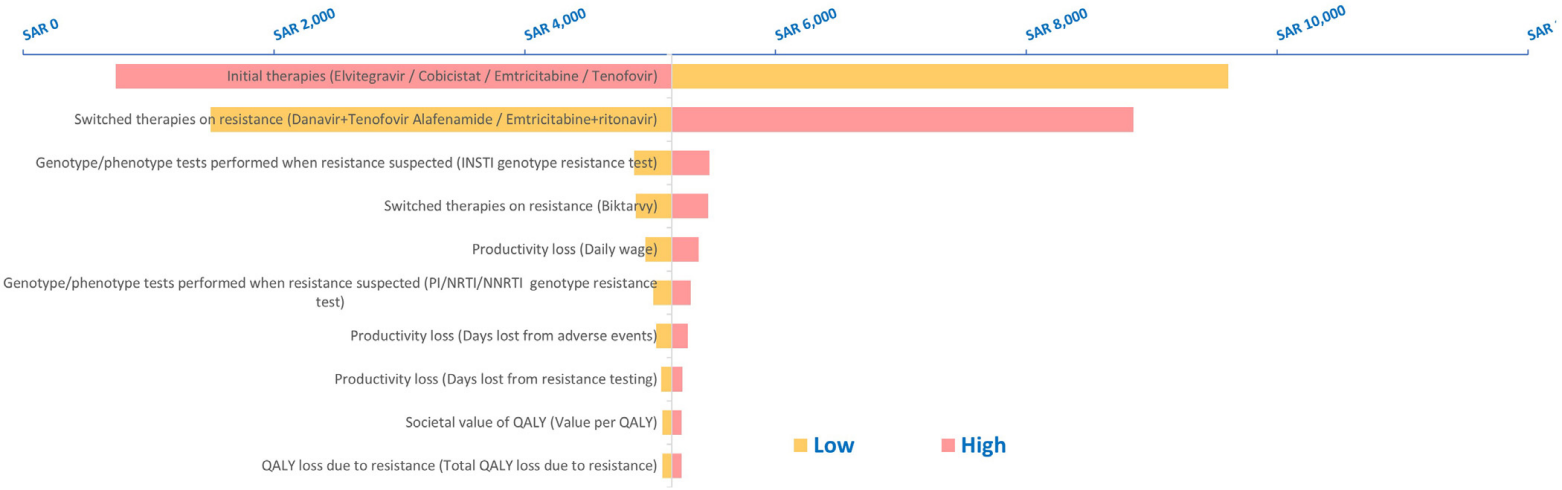
One-way sensitivity analysis for total costs of resistance, including ART costs per year.

ART, antiretroviral therapy; INSTI, integrase strand transfer inhibitor; NA, not applicable; NRTI, nucleoside reverse transcriptase inhibitor; NNRTI, non-NRTI; PI, protease inhibitor; QALY, quality-adjusted life-year; SAR, Saudi riyal.

In addition, a one-way sensitivity analysis was performed, wherein various input parameters were varied individually to study the impact on the outcomes of the study. The results, which was the total costs of resistance including ART costs per year, are presented as a tornado chart to present the effect of most impactful parameters, with 10% deviation in the model inputs (Figure 1).

All the model inputs and assumptions were validated at the final step by the experts.

## Results

### Direct costs

Per-patient direct health care costs were calculated based on the model inputs. The total direct cost included additional ART costs, additional clinic visits, cost for resistance tests, additional clinical tests for new ART, and for AEs from new ART (Table 4). The costs for AEs depended on the new ART that the patient switched to and costs to manage those AEs, as shown in Table 1, Table 4. Thus, the estimated per patient total direct health care costs without additional ART costs was Saudi Arabian riyal (SAR) 6980.

**Table 4:** Costs associated with HIV drug resistance per person in the year resistance develops.

Cost category	ART 1	ART 2	ART 3	ART 4
Direct healthcare cost (SAR)				
Annual additional ART costs	24,423	17,474	13,690	-4668
Additional clinic visits for resistance testing	300	300	300	300
Resistance tests	4500	4500	4500	4500
Additional clinical tests for new ART	2018	2018	2018	2018
Adverse events from new ART	162	162	162	162
**Total healthcare costs (year resistance develops)**	**31,403**	**24,453**	**20,669**	**2312**
Indirect cost (SAR)				
QALY loss	750	750	750	750
Productivity loss (resistance testing)	845	845	845	845
Productivity loss (adverse events)	1267	1267	1267	1267
**Total indirect costs**	**2862**	**2862**	**2862**	**2862**
***Total cost of virological failure per person developing resistance in 1 year***	**34,265**	**27,315**	**23,531**	**5174**

ART, antiretroviral therapy; QALY, quality-adjusted life-year; SAR, Saudi riyal.

ART 1: lamivudine/dolutegravir

ART 2: dolutegravir/lamivudine/abacavir

ART 3: dolutegravir + tenofovir alafenamide/emtricitabine

ART 4: elvitegravir/cobicistat/emtricitabine/tenofovir alafenamide

The annual per-patient costs of additional ART varied based on their first-line ART (Table 4). The patient may need to shift from low- to high-cost treatment, resulting in positive additional ART costs or may have to switch from high- to low-cost treatment, which resulted in negative additional ART cost. Total per-patient direct health care costs considering additional ART costs due to resistance ranged from SAR 2312 to 31,403.

### Indirect costs

The per-patient indirect costs were same for all initial four ARTs that included QALY loss and productivity loss due to resistance tests and AEs (Table 4).

### Total annual cost

The per-patient cost of additional ART varied based on their first-line ART. The total annual cost of virological failure per person developing resistance was calculated based on the direct and indirect costs and ranged from SAR 5174 to SAR 34,265.

### One-way sensitivity analysis

A one-way sensitivity analysis was used to analyze different model assumptions and their impact on the total costs due to resistance. Different parameters were studied, which included initial therapy used, switching therapy, resistance tests performed, productivity loss, and QALY loss. It was observed that initial therapies that were used by the patient and the therapies patients were switched to after resistance developed had a significant impact on the total costs of resistance per year, as shown in Figure 1.

## Discussion

This study evaluated the costs associated with an initial episode of drug resistance after first-line ART in KSA using a Microsoft Excel–based cost calculator for a patient.

Previous studies have reported development of resistance to at least one ART drug and, consequently, patients switching to new ARTs [[15], [16], [17]]. A retrospective study in Oman reported 83.7% of the patients with resistance to at least one drug and, after confirmation through resistance tests, 78% switched to new ART [16]. The WHO strongly recommends the use of resistance tests to prevent further spread of resistance [18]. A retrospective study conducted in the United States reported around 9% increase in health care costs for patients who switched ART compared with those who did not switch [19]. Owing to the lack of similar cost estimation studies in KSA, we compared our results with the cost estimation study that was conducted in Canada that considered primary and secondary resistance [9]. In this retrospective database study, the mean per-patient per-month costs increased by 31% after a positive resistance test. Moreover, the costs for one, two, or three ART class resistance were Canadian dollars 1278, 1337, and 1801, respectively [9]. The main driver of cost in ART-experienced patients was the cost due to new ART regimens, as also found in our analysis. Another study performed in the US showed that the mean per-patient follow-up cost increased from $70,000 for patients on first- or second-line treatment regimens to $105,130 for patients on third or later lines [20]. Our results indicated a substantial per-patient cost burden of SAR 5174-34,265 associated with initial episode of drug resistance including direct and indirect costs. The main cost drivers of direct health care expenditures were cost of additional ART regimen, followed by cost of resistance tests. The productivity loss due to AEs was the main cost driver of indirect expenditure.

However, there are a few limitations of this study. First, due to a paucity of available data for patients and their specific characteristics, the study estimated the total cost of ART drug resistance for one patient. Second, we did not estimate the additional cost implications due to increased risk of transmission during the time a patient is resistant to first-line ART. This could pose a public health threat and may add to the existing economic burden. Third, our study only focused on primary resistance and did not include a patient population with secondary or acquired resistance. Fourth, some model input parameters were based on expert opinion owing to a lack to country-specific data and therefore could be subject to bias.

Significant cost burden due to resistance to ART, as observed in our study, emphasizes the selection of appropriate initial treatment strategy to mitigate the associated clinical and economic burden.

## Conclusion

There is a substantial cost burden for individuals who develop resistance after first-line ART. The cost of additional ART and resistance tests are the main cost drivers. Offering regimens with strong genetic and pharmacologic resistance barriers could help reduce the risk of resistance and the associated costs. The study emphasizes need of further studies to analyze cost burden due to the transmission of virus in KSA, which would help in formulating better health care policies and guidelines for the treatment of patients with HIV.

## Declarations of competing interest

Asma Mestouri and Rezk Elaraby are employees of Gilead, Dubai, UAE. The remaining authors have no competing interests to disclose.

## Appendix Supplementary materials

Image, application 1
