# Supplementary material for: Estimating the cost due to resistance against antiretroviral therapies in individuals with HIV: Perspective of the Kingdom of Saudi Arabia
Source: IJID Reg. 2024 Apr 25;11:100371. doi: 10.1016/j.ijregi.2024.100371 (PMC11130718; doi:10.1016/j.ijregi.2024.100371)
Supplement: Supplementary file 1 [file mmc1.docx]

**Supplementary Document**

**Title of the article:** Estimating the Cost Due to Resistance Against Antiretroviral Therapies in Individuals with HIV: A National perspective of the Kingdom of Saudi Arabia

**Running title:** Economic Burden of ART Resistance in PLWHIV: KSA

Supplementary Table S1: Key features of the model

| Element | | Definition |
| --- | --- | --- |
| Perspective | Societal | |
| Model Type | Cost calculator developed in MS Excel | |
| Currency | SAR | |
| Time Horizon | One year for costs associated with testing for resistance, tests required for a new ART regimen, costs of adverse events of a new regimen and indirect costs. | |
| Population | Adults with HIV experiencing 1st episode of drug resistance | |
| Key Inputs | - Clinical visits - Proportion of patients requiring resistance tests - Cost of resistance tests - Cost of additional clinical costs due to HIV resistance - Adverse event rates - Resources used of managing adverse events - Cost of healthcare resources - Cost of ARTs - QALY loss due to resistance - Productivity loss | |
| Key Outputs | Cost per patient per episode of drug resistance, including the cost of new ART in one year | |

ART, antiretroviral therapy; HIV, human immunodeficiency virus; QALY, quality-adjusted life-years; SAR, Saudi riyals

Supplementary Table S2: Percentage of Patients Switching to Different Regimens Upon Developing Resistance

| Initial regimen | Bictegravir / emtricitabine / tenofovir alafenamide | Tenofovir alafenamide + ritonavir + darunavir + dolutegravir | Darunavir + tenofovir alafenamide/ emtricitabine + ritonavir | Source |
| --- | --- | --- | --- | --- |
| Lamivudine / dolutegravir | 10% | 90% | 0% | KOL Inputs |
| Dolutegravir / lamivudine / abacavir | 10% | 90% | 0% | KOL Inputs |
| Dolutegravir + tenofovir alafenamide / emtricitabine | 10% | 90% | 0% | KOL Inputs |
| Elvitegravir / cobicistat / emtricitabine / tenofovir alafenamide | 10% | 0% | 90% | KOL Inputs |

KOL, key opinion leader

Supplementary Table S3: Costs Associated With HIV Drugs

| Therapies | Cost per year (CIF) | Source |
| --- | --- | --- |
| Initial regimens |  |  |
| Lamivudine / dolutegravir | SAR 29,030 | Summary of Product Characteristics (SmPC) and SFDA |
| Dolutegravir / lamivudine / abacavir | SAR 35,980 | SmPC and SFDA |
| Dolutegravir + tenofovir alafenamide / emtricitabine | SAR 39,764 | SmPC and SFDA |
| Elvitegravir / cobicistat / emtricitabine / tenofovir alafenamide | SAR 44,370 | SmPC and SFDA |
| Switched therapies upon developing resistance |  |  |
| Tenofovir alafenamide | SAR 9,966 | SmPC and SFDA |
| Ritonavir | SAR 15,006 | SmPC and SFDA |
| Darunavir | SAR 8,660 | SmPC and SFDA |
| Dolutegravir | SAR 22,538 | SmPC and SFDA |
| Tenofovir alafenamide + ritonavir + darunavir + dolutegravir | SAR 56,171 | SmPC and SFDA |
| Ritonavir + darunavir | SAR 23,667 | SmPC and SFDA |
| Darunavir + tenofovir alafenamide / emtricitabine + ritonavir | SAR 40,892 | SmPC and SFDA |
| Bictegravir / emtricitabine / tenofovir alafenamide | SAR 28,998 | SmPC and SFDA |

CIF, Cost insurance and freight; HIV, human immunodeficiency virus; SAR, Saudi riyals; SFDA, Saudi Food and Drug Authority

Supplementary Table S4: Rates for Adverse Events

| Adverse Event | Rates |
| --- | --- |
| **Patients on Darunavir + Tenofovir alafenamide/ Emtricitabine +Ritonavir ^a^** | |
| Gastrointestinal | 8% |
| Alanine aminotransferase | 13% |
| Aspartate aminotransferase | 13% |
| Total cholesterol | 24% |
| LDL elevation | 23% |
| Hyperglycemia | 12% |
| Pancreatic amylase | 9% |
| Diarrhoea | 5% |
| Triglycerides | 6% |
| **Patients on Tenofovir alafenamide + Ritonavir + Darunavir + Dolutegravir ^a^** | |
| Gastrointestinal | 8% |
| Alanine aminotransferase | 13% |
| Aspartate aminotransferase | 13% |
| Total cholesterol | 24% |
| LDL elevation | 23% |
| Hyperglycemia | 12% |
| Pancreatic amylase | 9% |
| Diarrhoea | 5% |
| Triglycerides | 6% |
| Patients on Emtricitabine + Bictegravir + Tenofovir alafenamide ^b^ | |
| Diarrhoea | 12% |
| Back pain | 6% |
| Nausea | 7.7% |
| Arthralgia | 6.2% |
| Fatigue | 6.2% |
| Headache | 7.7% |
| Chlamydial infection | 6.2% |
| Upper respiratory tract infection | 7.7% |
| Creatine kinase concentration elevation | 12.5% |
| AST elevation | 9.4% |
| Serum glucose concentration elevation (fasting hyperglycaemia) | 7.8% |
| ALT elevation | 6.3% |
| LDL elevation | 6.3% |

ALT, alanine aminotransferase; AST, aspartate aminotransferase; LDL, low-density lipoprotein

Sources: a, Orkin C et al. Final 192-week efficacy and safety of once-daily darunavir/ritonavir compared with lopinavir/ritonavir in HIV-1-infected treatment-naïve patients in the ARTEMIS trial. *HIV Med*. 2013;14(1):49-59.

b, Sax, P. E. et al. Bictegravir versus dolutegravir, each with emtricitabine and tenofovir alafenamide, for initial treatment of HIV-1 infection: a randomised, double-blind, phase 2 trial. *The lancet. HIV. 2017*, *4*(4), e154–e160.

Supplementary Table S5: Drugs Used for Adverse Events and Their Costs

| AEs | Prescribed Drugs | Dosage | Dose Per Day | Duration of Treatment (Days) | Cost per Dose (SAR)^*^ | Source for Dose and Duration |
| --- | --- | --- | --- | --- | --- | --- |
| Headache | Paracetamol | 500 mg | 3 | 2 | 0.05 | KOL |
|  | Ibuprofen | 400 mg | 3 | 2 | 0.11 | KOL |
| Dizziness | None | None | 0 | 0 | - | KOL |
| Peripheral neuropathy | Pregabalin | 300 mg | 1 | 28 | 4.09 | KOL (assumption on dosage and duration) |
| Nausea | Metoclopramide | 10 mg | 2 | 28 | 0.24 | KOL (assumption on duration) |
|  | Ondansetrone | 4 mg | 2 | 28 | 7.98 | KOL (assumption on duration) |
| Diarrhoea | Loperamide | 10 mg | 2 | 2 | 0.65 | KOL (assumption on duration) |
| Back pain | Pregabalin | 300 mg | 1 | 28 | 4.09 | Assumed same as for neuropathy |
| ALT elevation | No treatment | None | 0 | 0 | - | KOL |
| AST elevation | No treatment | None | 0 | 0 | - | KOL |
| Fatigue | No treatment | None | 0 | 0 | - | KOL |
| Amylase elevations | Tazocin | 4.5 gm | 3 | 7 | 55.61 | KOL, SmPC |
|  | IV Fluids | 500 mL | 5 | 7 | 5.00 | KOL |
| Gastrointestinal | Hyoscine | 10 mg | 2 | 2 | 0.27 | IQVIA Insights |
| Alanine aminotransferase | No treatment | None |  |  | 0.00 |  |
| Aspartate aminotransferase | No treatment | None |  |  | 0.00 |  |
| Total cholesterol | Atorvastatin | 40 mg | 1 | 180 | 1.27 | IQVIA Insights |
| LDL elevation | Atorvastatin | 40 mg | 1 | 180 | 1.27 | IQVIA Insights |
| Hyperglycemia | Metformin | 500 mg | 2 | 30 | 0.18 | IQVIA Insights (30 days assumed for per episode of hyper gycemia) |
|  |  |  |  |  |  |  |
| Triglycerides | Atorvastatin | 40 mg | 1 | 180 | 1.27 | IQVIA Insights |
|  | Fenofibrate | 200 mg | 1 | 180 | 0.92 | IQVIA Insights |
| Arthralgia | Ibuprofen | 400 mg | 2 | 7 | 0.11 | IQVIA Insights (7 days assumed for episode of arthralgia) |
| Chlamydial infection | Doxycycline | 100 mg | 2 | 7 | 2.15 | WHO guidelines (Azithromycin 1gm single dose OR Doxycycline for 7 days) |
| Upper respiratory tract infection | Azithromycin | 500 mg | 1 | 3 | 7.80 | IQVIA insights (Azithromycin 500 mg once a day for 3 days, paracetamol 500 mg thrice a day for 3 days) |
|  | Paracetamol | 500 mg | 3 | 3 | 0.05 |  |
| Creatine kinase concentration elevation | No treatment | None |  |  |  |  |
| Serum glucose concentration elevation (fasting hyperglycaemia) | Metformin | 500 mg | 2 | 30 | 0.18 | Assumed 1 month of treatment per episode |

AEs, adverse events; ALT, alanine aminotransferase; AST, aspartate aminotransferase; IV, intravenous; gm, gram; KOL, key opinion leader; mg, milligram; NA, not applicable; SAR, Saudi riyals; SFDA, Saudi Food and Drug Authority; SmPC, Summary of Product Characteristic

^*^ All drug costs were from SFDA
